# Study of the Association between *ITPKC* Genetic Polymorphisms and Calcium Nephrolithiasis

**DOI:** 10.1155/2014/397826

**Published:** 2014-03-03

**Authors:** Wei-Chih Kan, Yii-Her Chou, Siou-Jin Chiu, Yu-Wen Hsu, Hsing-Fang Lu, Wenli Hsu, Wei-Chiao Chang

**Affiliations:** ^1^Department of Nephrology, Chi-Mei Medical Center, Tainan 710, Taiwan; ^2^Medical Laboratory Science and Biotechnology, Chung Hwa University of Medical Technology, Tainan 717, Taiwan; ^3^Department of Urology, College of Medicine, Kaohsiung Medical University, Kaohsiung 807, Taiwan; ^4^Department of Clinical Pharmacy, Taipei Medical University, Taipei 110, Taiwan; ^5^Cancer Center, Kaohsiung Medical University Hospital, Kaohsiung 807, Taiwan; ^6^Department of Clinical Medicine, Kaohsiung Medical University, Kaohsiung 807, Taiwan; ^7^Master Program for Clinical Pharmacogenomics and Pharmacoproteomics, School of Pharmacy, Taipei Medical University, Taipei 110, Taiwan; ^8^Department of Pharmacy, Taipei Medical University, Wanfang Hospital, Taipei 116, Taiwan

## Abstract

Nephrolithiasis is a multifactorial disease caused by environmental, hormonal, and genetic factors. Genetic polymorphisms of *ORAI1*, which codes for the main subunit of the store-operated calcium (SOC) channel, were reported to be associated with the risk and recurrence of calcium nephrolithiasis. Inositol 1,4,5-trisphosphate (IP3) 3-kinase C (ITPKC) is a negative regulator of the SOC channel-mediated signaling pathway. We investigated the association between calcium containing nephrolithiasis and genetic variants of *ITPKC* gene in Taiwanese patients. 365 patients were recruited in this study. Eight tagging single nucleotide polymorphisms of *ITPKC* were selected for genotyping. *ITPKC* genotypes were determined by TaqMan assay. *ITPKC* plasmids were transfected into cells to evaluate the intracellular calcium mobilization. Our results indicated that rs2607420 CC genotype in the intron region of the *ITPKC* gene is associated with a lower eGFR by both Modification of Diet in Renal Diseases (*P* = 0.0405) and Cockcroft-Gault (*P* = 0.0215) equations in patients with calcium nephrolithiasis. Our results identify a novel polymorphism for renal function and highlight the importance of ITPKC as a key molecule to regulate calcium signaling.

## 1. Introduction

Urolithiasis is a global problem affecting almost all populations in the world. In developed countries, the prevalence rate of urolithiasis was reported to be 4%~20%. In Taiwan, the prevalence was reported to be 9.6% [1]. The lifetime risk of urolithiasis is about 10%~15% in the developed world, but the risk was as high as 20%~25% in the Middle East [2]. Furthermore, in 20%~75% of patients, the disease recurs within 10 years of the first episode [3]. Consequently, urolithiasis causes a burden on society and significantly influences patients' quality of life. Previous epidemiological studies described an association between obesity and nephrolithiasis [4].

Urolithiasis often involves the formation of stones containing calcium compounds, mainly calcium oxalate and calcium phosphate, which account for 70%~80% of reported cases of urolithiasis. Calcium urolithiasis is thought to have a physicochemical origin, involving processes such as nucleation, growth, aggregation, and retention of crystals in the urine. The crystals include inorganic (e.g., calcium, uric acid, phosphate, and citrate) and organic substances (the Tamm-Horsfall glycoprotein and osteopontin) [5]. Calcium nephrolithiasis is a type of calcium metabolism disorder. Several studies indicated that the crystals may injure renal epithelial cells through inflammatory reactions and apoptosis, resulting in stone formation [6–8]. It is thought to be a multifactorial disease influenced by environmental, hormonal, and genetic factors.

Our previous study indicated that genetic polymorphisms of *ORAI1*, which codes for the main subunit of the store-operated calcium (SOC) channel, were associated with the risk and recurrence of calcium nephrolithiasis in a Taiwanese population [9]. Inositol 1,4,5-trisphosphate (IP_3_) 3-kinase C (ITPKC) is a negative regulator of the SOC channel-mediated nuclear factor of activated T cell (NFAT) signaling pathway and is involved in immune modulation via phosphorylation of IP_3_ [10, 11]. A functional single-nucleotide polymorphism (SNP) of *ITPKC* (rs28493229) was found to be associated with susceptibility to Kawasaki disease and coronary artery lesion formation [11]. The purpose of this study was to determine whether SNPs (rs11673492, rs28493229, rs7257602, rs7251246, rs890934, rs10420685, rs2607420, and rs2290692) of* ITPKC* are associated with the *recurrence,* stone number, or kidney function of patients with nephrolithiasis.

## 2. Material and Methods

### 2.1. Patients and Methods

We enrolled 365 patients who fulfilled the diagnostic criteria for nephrolithiasis at Kaohsiung Medical University Hospital (KMUH). Radiographic and echographic documentation of urinary stones in these patients were collected. Stone samples were obtained either from spontaneous passage or by surgical manipulation. We also collected clinical information such as age, gender, family history of nephrolithiasis, and episodes of stone recurrence. The past history of the stone episode was traced back to the whole life as far as patients could remember. Patients with at least 2 symptomatic episodes (at least 6 months apart) or new stones after treatment were classified into the recurrent group, and those with only 1 episode were classified into the single group. All subjects provided informed consent. The study protocol conformed to the *Declaration of Helsinki* and the study was approved by the Institute Review Board of KMUH.

### 2.2. DNA Extraction

DNA was extracted from blood samples collected from subjects. Blood cells were first treated with 0.5% sodium dodecyl sulfate lysis buffer and then with protease K (1 mg/mL) for 4 h at 60°C to digest the nuclear proteins. Total DNA was harvested using a Gentra (QIAGEN Inc., Valencia, CA) extraction kit and 70% alcohol precipitation, as in our previous study [12].

### 2.3. Genotyping

**Figure 1:**
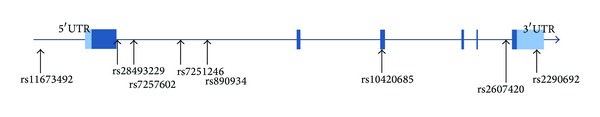
Graphical overview of the genotyped human *ITPKC* gene.

We selected seven tagging SNPs (rs11673492, rs7257602, rs7251246, rs890934, rs10420685, rs2607420, and rs2290692) with a minor allele frequency greater than 10% in the Han Chinese Beijing population from the HapMap database (http://hapmap.ncbi.nlm.nih.gov/). In addition, we included rs28493229 in this study which resulted from its association with the inflammation which has been demonstrated. The *ITPKC* gene structure was shown in Figure 1. Genotyping was carried out using the TaqMan allelic discrimination assay (Applied Biosystems, Foster City, CA). Briefly, we performed a polymerase chain reaction (PCR) using a 96-well microplate with an ABI 9700 thermal cycler (Applied Biosystems). The thermal cycle conditions were as follows: denaturing at 95°C for 10 min, followed by 40 cycles of denaturing at 92°C for 15 s, and annealing and extending at 60°C for 1 min. After the PCR, the fluorescence was measured and analyzed using System SDS software version 1.2.3 (Applied Biosystems, Foster City, CA).

### 2.4. Calcium Imaging

Intracellular Ca^2+^ ([Ca^2+^]_*i*_) responses were induced by application of thapsigargin (TG) (Sigma-Aldrich, St. Louis, MO) in ITPKC-overexpressing HEK293 cells, according to the previously described methods [13]. Before the experiments, cells were stained with 1 *μ*M Fluo-4-AM (Molecular Probes, Eugene, OR) at 37°C for 20 min and then washed with BSS buffer (5.4 mM KCl, 5.5 mM D-glucose, 1 mM MgSO_4_, 130 mM NaCl, 20 mM Hepes at pH 7.4, and 2 mM CaCl_2_). [Ca^2+^]_*i*_ concentrations were estimated based on the ratio of fluorescence intensities emitted upon excitation with consecutive 3-s pulses of 488 nm light at a resolution of 1376 × 1038 pixels using an Olympus Cell ^∧^R IX81 fluorescence microscope (Olympus, Essex, UK) equipped with an MT 20 illumination system (Olympus) and UPLanApo 10x objective lens. The [Ca^2+^]_*i*_ concentration was estimated based on calibration curves as follows. A Ca^2+^ calibration curve was created using a Ca^2+^ calibration buffer kit (Molecular Probes). This experiment was repeated five times. [Ca^2+^]_*i*_ was calculated from Fluo-4 excited at 488 nm and imaged using an Olympus Cell ^∧^R IX81 fluorescence microscope and UPLanApo 10x objective lens at 20°C. Fluo-4 signals were calibrated by measuring the fluorescence intensity from microcuvettes containing 10 mM K_2_-EGTA (pH 7.20) buffered to various [Ca^2+^]_*i*_ levels. The Ca^2+^ concentration was calculated using the following formula: [Ca^2+^]_*i*_ = KD × ((*F* − *F*_min⁡_)/(*F*_max⁡_ − *F*)). Plotting the fluorescence intensity versus [Ca^2+^]_i_ yielded the calibration curve with the formula of [Ca^2+^]_*i*_ = KD × ((*F* − *F*_min⁡_)/(*F*_max⁡_ − *F*)), where KD is 345 nM, *F* is the Fluo-4 intensity, *F*_max⁡_ is 640, and *F*_min⁡_ is 21.7 for Fluo-4.

### 2.5. Statistical Analysis

SAS 9.1 for Windows (Cary, NC) was used for all statistical analyses. Statistical differences in genotypes and allelic frequencies between cases and controls were assessed by *χ* ^2^ test. A *P*  value of <0.05 was considered to be statistically significant.

## 3. Results

### 3.1. Demographic and Clinical Characteristics of Subjects

We included 365 nephrolithiasis patients in this study.  Table 1 shows the demographic characteristics of subjects. The mean age of patients ± standard deviation was 54.3 ± 12.0 years, and 68.5% of patients were male (Table 1). In total, 8 tagging SNPs of *ITPKC* were selected in this study from the HapMap database (http://hapmap.ncbi.nlm.nih.gov/). These SNPs had at least 10% minimum allele frequencies (MAFs) in a Han Chinese Beijing population. A graphical overview of the genotyped polymorphisms is shown in Figure 1.

### 3.2. Association Study of *ITPKC* Genetic Polymorphisms with the Risk of Stone Numbers and Recurrence in Patients with Calcium Nephrolithiasis

Among cases, 113 patients had had recurrent episodes, and 116 patients had had a single episode. We tested whether the rs11673492, rs28493229, rs7257602, rs7251246, rs890934, rs10420685, rs2607420, and rs2290692 genotypes were associated with the stone number or recurrence of calcium nephrolithiasis. As shown in Table 2, we found no significant association of genotypes or allelic frequencies with stone numbers. No significant association was noted between genotypes and the recurrence of calcium nephrolithiasis (Table 2).

### 3.3. Associations of *ITPKC* Genetic Polymorphisms with the Estimated Glomerular Filtration Rate (eGFR)

Previous studies indicated that renal stones may cause renal impairment and decrease renal function [14]. Therefore, we further calculated Modification of Diet in Renal Diseases- (MDRD-) based and Cockcroft-Gault- (C-G-) based eGFRs which are widely used tools to predict the renal function of nephrolithiasis patients [15, 16]. We tested the relationship between *ITPKC* genetic polymorphisms and renal function. Among the SNPs of *ITPKC* we tested, rs7251246 had a significant association (*P* = 0.0456) with a lower eGFR as calculated by C-G, while the rs2607420 CC genotype had a significant correlation with a lower eGFR as calculated by both MDRD (*P* = 0.0405) and C-G (*P* = 0.0215) (Table 3).

### 3.4. Effects of ITPKC Overexpression in Calcium Signaling in HEK293 Cells

**Figure 2:**
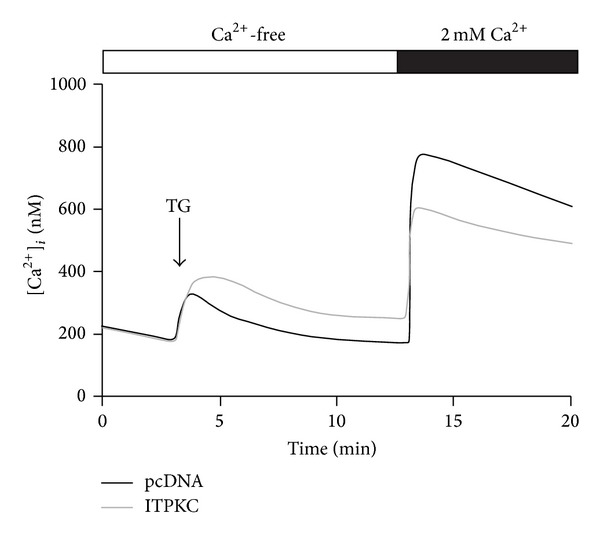
Effects of ITPKC on calcium influx in HEK293 cells. Thapsigargin (TG; 2 *μ*M) was applied in a Ca^2+^-free BSS solution. The extracellular Ca^2+^ concentration abruptly increased from 0 to 2 mM which triggered the store-operated (SOC) channel influx.

The mechanism by which ITPKC influences cellular signaling is not clear. Thus, we transfected ITPKC plasmids into cells and evaluated the intracellular calcium mobilization. As shown in Figure 2, overexpression of ITPKC slightly increased the calcium release (first calcium peak). Importantly, the sustained calcium influx was reduced (second calcium peak).

## 4. Discussion

Nephrolithiasis is caused by a variety of conditions, including metabolic disorders and anatomical defects. It is considered a metabolic disorder commonly associated with type 2 diabetes, obesity, dyslipidemia, and hypertension. Most cases of nephrolithiasis are idiopathic; in such cases, there is undoubtedly a genetic predisposition, but both environmental and lifestyle factors may play important roles [17]. Almost 40% of patients who develop a stone for the first time will develop a second stone within 3 years of the first episode if prophylactic measures are not taken. The physicochemical theory of stone formation considers urine a supersaturated solution in which homogeneous or heterogeneous nucleation can lead to initiation of crystal formation, which can then aggregate and grow into a stone [17]. A well-known theory is Randall's plaques; this theory proposes that subepithelial interstitial calcium-based deposits act as nuclei for stone formation. These plaques, which are composed of apatite (calcium phosphate, not calcium oxalate), originate adjacent to the thin limbs of the loops of Henle. As they grow in size, they may injure the renal papillary duct epithelium and serve as sites for intratubular adhesion and growth of calcium oxalate crystals [17]. A positive family history of kidney stones is strongly associated with an increased risk of stone formation; the relative risk is 2~3 times higher in such individuals than in individuals without a family history of kidney stones [6].

Calcium nephrolithiasis is a type of calcium metabolic disorder, and most renal stones contain calcium. Formation of stones may also result from injury and inflammation of the renal tubular epithelium. Recent study indicated that crystals deposition or formation inside the kidney not only directly causes renal epithelial cell damage but also may induce immune response [18]. Crystals act as a stimulant that trigger innate immunity and lead to generate several cytokines such as IL-1*β* and IL-18 that drive inflammatory response [18]. Genetic factors that regulate calcium metabolism and inflammation are possibly linked with nephrolithiasis. Genetic polymorphisms of *CLDN14, CASR, OPN, ORAI1,* and *VDR* were reported to be involved in calcium nephrolithiasis in humans [19–23]. Our previous study indicated that genetic polymorphisms of *ORAI1* are associated with susceptibility to calcium urolithiasis [6]. ORAI1 is a pore subunit of the SOC channel involved in different physiological functions, including immune responses and inflammatory reactions [24]. Indeed, calcium influx through the SOC channel can regulate the secretion of proinflammatory molecules such as arachidonic acid and leukotriene C_4_ [25]. *ITPKC* is an upstream gene of the SOC channel that may influence its activation via IP_3_ phosphorylation, which in turn regulates immune responses [26]. Therefore, we supposed that genetic variant of *ITPKC* may contribute to influence the calcium influx and, furthermore, may induce the activation of T cells which provoke inflammatory reaction. By using fluorescence-based calcium detection, we provide the first evidence to support a functional role of ITPKC in calcium signaling (Figure 2).

In this study, we found no significant associations between genotypes of *ITPKC* (rs11673492, rs28493229, rs7257602, rs7251246, rs890934, rs10420685, rs2607420, and rs2290692) and calcium nephrolithiasis. However, we cannot rule out the possibility that other low-frequency genetic polymorphisms of *ITPKC* may contribute to calcium nephrolithiasis. Importantly, we found that rs2607420 was significantly associated with kidney function (MDRD and C-G). Although we had no evidence to prove that rs2607420, the intronic SNP, directly affects the splicing efficiency or influences the affinity of transcription factor binding site, however, another intronic SNP of *ITPKC*, rs28493229, had demonstrated that the variant of rs28493229 is associated with the mRNA expression level of *ITPKC* [11]. Thus, the variant of rs2607420 may result in enhancing the risk of renal injury through changing *ITPKC *expression level. Even so, the effects of ITPKC on calcium nephrolithiasis and kidney functions are still unclear. More research is needed to determine the molecular basis of *ITPKC* and the pathogenesis of calcium nephrolithiasis.

In conclusion, we found that rs2607420 in the intron region of the ITPKC gene is associate with estimated creatinine clearance in patients with calcium nephrolithiasis.

## Figures and Tables

**Table 1:** Basal characteristics of patients with nephrolithiasis and of normal controls.

Characteristic	Patients with nephrolithiasis
Number of subjects	365
Gender: male, number (%)	250 (68.5%)
Age (years)^a^	54.3 ± 12.0
Range	24~86

^a^Mean ± SD.

**Table 2:** Association analysis of ITPKC single-nucleotide polymorphisms (SNPs) and clinical medical records data of patients with kidney stones.

SNP	Genotype	Stone numbers (%)		*P* value	Stone frequency (%)		*P* value
		Multiple	Single		Recurrence	Nonrecurrence	
rs11673492	TT	12 (9.7)	10 (8.1)	0.9050	11 (9.7)	10 (8.7)	0.4025
	CT	58 (46.8)	59 (47.6)		46 (40.7)	57 (49.6)	
	CC	54 (43.5)	55 (44.3)		56 (49.6)	48 (41.7)	

rs28493229	CG	18 (14.8)	10 (8.1)	0.1032	16 (14.4)	9 (7.9)	0.1198
	GG	104 (85.2)	113 (91.9)		95 (85.6)	105 (92.1)	

rs7257602	GG	32 (25.8)	24 (19.2)	0.3705	27 (23.9)	23 (19.8)	0.7665
	AG	55 (44.4)	65 (52.0)		54 (47.8)	58 (50.0)	
	AA	37 (29.8)	36 (28.8)		32 (28.3)	35 (30.2)	

rs7251246	GG	38 (30.7)	30 (24.0)	0.2634	29 (25.7)	33 (28.5)	0.7521
	GA	50 (40.3)	63 (50.4)		52 (46.0)	55 (47.4)	
	AA	36 (29.0)	32 (25.6)		32 (28.3)	28 (24.1)	

rs890934	TT	23 (18.5)	29 (23.2)	0.3919	23 (20.3)	24 (20.7)	0.9975
	GT	58 (46.8)	62 (49.6)		55 (48.7)	56 (48.3)	
	GG	43 (34.7)	34 (27.2)		35 (31.0)	36 (31.0)	

rs10420685	GG	7 (5.6)	5 (4.0)	0.7842	6 (5.3)	6 (5.2)	0.8333
	AG	43 (34.7)	41 (33.1)		37 (32.7)	42 (36.5)	
	AA	74 (59.7)	78 (62.9)		70 (62.0)	67 (58.3)	

rs2607420	CC	10 (8.1)	9 (7.2)	0.9618	10 (8.9)	9 (7.8)	0.9191
	CT	51 (41.5)	52 (41.6)		43 (38.4)	47 (40.5)	
	TT	62 (50.4)	64 (51.2)		59 (52.7)	60 (51.7)	

rs2290692	GG	38 (30.7)	28 (22.4)	0.2161	28 (24.8)	32 (27.6)	0.8602
	CG	50 (40.3)	63 (50.4)		53 (46.9)	54 (46.5)	
	CC	36 (29.0)	34 (27.2)		32 (28.3)	30 (25.9)	

**Table 3:** Association analysis of ITPKC single-nucleotide polymorphisms (SNPs) and clinical biochemical data of patients with kidney stones.

SNP	Genotype	Sample number (%)	MDRD	*P* value	C-G	*P* value
			(mL/min/1.73 m^2^)		(mL/min)	
rs11673492	TT	32 (8.8)	77.6 ± 16.0	0.1471	71.4 ± 19.2	0.2342
	CT	164 (45.1)	84.9 ± 29.9		85.1 ± 33.0	
	CC	168 (46.1)	84.0 ± 25.9		83.0 ± 28.2	

rs28493229	CC	0 (0.0)	—	0.5622	—	0.8885
	CG	42 (12.5)	82.9 ± 26.4		83.2 ± 29.3	
	GG	295 (87.5)	86.9 ± 34.3		82.1 ± 36.7	

rs7257602	GG	92 (25.3)	80.5 ± 28.5	0.2855	77.9 ± 29.2	0.4558
	AG	164 (45.0)	86.8 ± 28.3		85.6 ± 29.3	
	AA	108 (29.7)	80.1 ± 24.7		82.3 ± 31.2	

rs7251246	GG	96 (26.4)	82.5 ± 22.4	0.0990	84.3 ± 28.9	0.0456*
	GA	164 (45.0)	87.6 ± 29.9		87.4 ± 30.7	
	AA	104 (28.6)	76.7 ± 27.3		73.2 ± 28.3	

rs890934	TT	77 (21.1)	77.9 ± 29.0	0.4380	74.4 ± 23.3	0.1932
	GT	180 (49.3)	84.5 ± 28.9		84.0 ± 32.7	
	GG	108 (29.6)	84.4 ± 23.6		86.0 ± 29.1	

rs10420685	GG	16 (4.4)	90.9 ± 12.5	0.2153	89.5 ± 21.1	0.1545
	AG	126 (34.6)	86.5 ± 26.4		87.7 ± 31.7	
	AA	222 (61.0)	80.2 ± 28.5		78.9 ± 29.3	

rs2607420	CC	27 (7.4)	69.0 ± 16.4	0.0405*	68.6 ± 18.2	0.0215*
	CT	143 (39.3)	87.2 ± 26.6		89.2 ± 31.6	
	TT	194 (53.3)	81.6 ± 27.9		79.2 ± 28.3	

rs2290692	GG	93 (19.4)	83.1 ± 22.6	0.4016	84.8 ± 29.3	0.0784
	CG	164 (34.2)	85.8 ± 27.9		86.7 ± 30.8	
	CC	107 (46.4)	78.9 ± 30.9		74.1 ± 28.5	

*Significant (*P* < 0.05) values are in bold.
